# Microsomal cytochrome P-450-linked monooxygenase systems and lipid composition of human hepatocellular carcinoma.

**DOI:** 10.1038/bjc.1989.3

**Published:** 1989-01

**Authors:** I. Hamamoto, S. Tanaka, T. Maeba, K. Chikaishi, Y. Ichikawa

**Affiliations:** Department of Surgery, Kagawa Medical School, Japan.

## Abstract

**Images:**


Br. J. Cancer (1989), 59, 6–11 © The Macmillan Press Ltd., 1989

### Microsomal cytochrome P-450-linked monooxygenase systems and lipid composition of human hepatocellular carcinoma

I. Hamamoto, S. Tanaka, T. Maeba, K. Chikaishi & Y. Ichikawa[1]

*Departments of Surgery and [1]Biochemistry, Kagawa Medical School, Miki-cho, Kita-gun, Kagawa 761-07, Japan*

**Summary** The tissues of hepatocellular carcinoma were operatively resected from six patients. All four components of the systems of microsomal cytochrome P-450-linked monooxygenase of the tissues were investigated and compared to those of normal liver tissue. The concentrations of cytochromes P-450, P-420 and $b_5$ were measured optically and the concentrations of all components except cytochrome P-450 were measured by the Western blotting method followed by immunochemical staining. In microsomes of hepatocellular carcinoma tissues, there was as much cytochrome P-450 and other redox components as in the normal liver tissues, but cytochrome P-450 in liver cancer tissues was unstable and easily converted to cytochrome P-420. The specific activities of NADPH- and NADH-ferricyanide and cytochrome c reductase of each sample were also measured. In the microsomes of the cancer tissues, the specific activities were remarkably reduced compared with those of normal liver tissues. The lipid compositions of the microsomes and the phospholipid/cholesterol ratios (w/w) were $13.1 \pm 3.13$ in the cancer tissues and $43.0 \pm 6.74$ in normal liver tissues. This difference of the lipid composition elucidates the instability of cytochrome P-450 molecules and the inefficiency of the electron transport of cytochrome P-450-linked monooxygenase systems.

The microsomal cytochrome P-450-linked monooxygenase system of the liver is important in drug metabolism (Cooper *et al.*, 1965) and carcinogenesis (Norman *et al.*, 1979). The system contains five components: NADPH-cytochrome P-450 reductase, NADPH-cytochrome $b_5$ reductase, cytochrome $b_5$, desaturase and cytochrome P-450 (Iyanagi & Mason, 1973; Hrycay & Prough, 1974). In human liver microsomes, there are multiple molecular forms of cytochrome P-450, some of which have been isolated and purified (Wrighton *et al.*, 1986; Wang *et al.*, 1983).

Although considerable information is available about the influence of chemically induced hyperplastic tumours on drug-metabolising enzymes in laboratory animals (Eriksson *et al.*, 1983; Astrom *et al.*, 1983), there is little information on changes in these enzymes induced in human liver by neoplasia such as hepatocellular carcinoma. In the preneoplastic stage of chemically induced hepatic nodules of rats, these drug-oxidising enzymes are decreased (Farbe, 1984), but in human hepatic neoplasms there is no report on the enzyme molecules of the microsomal cytochrome P-450-linked monooxygenase systems except for their activities (El Mouelhi *et al.*, 1987).

The absorption at 450 nm of reduced cytochrome P-450 under carbon monoxide is unusual among the known b-type cytochromes, but the electron paramagnetic resonance absorption of this cytochrome is typical and easily detectable (Mason *et al.*, 1965). Some workers have reported the electron paramagnetic resonance spectra of the high-spin or low-spin forms of cytochrome P-450 in microsomes or purified cytochromes P-450 of animals (Ichikawa & Yamano, 1970; Ichikawa *et al.*, 1967). No electron paramagnetic resonance spectrum of the intact tissues of normal human liver or hepatic neoplasm has been reported. The electron paramagnetic resonance spectra of normal liver and hepatocellular carcinoma tissues of humans were measured.

In many species, the lipid compositions of hepatic microsomes have been measured to predict the properties of the lipid bilayers; the molar ratios of phospholipid to cholesterol in human liver microsomes have been estimated to be 14.5 (Waskell *et al.*, 1982) and 7.5 (Kapitulnik *et al.*, 1987), and the weight ratio to be 30.9 (Benga *et al.*, 1983). But the relationship between the change of the lipid composition and carcinogenesis has not been clarified.

Correspondence: I. Hamamoto.
Received 5 May 1988; and in revised form, 30 July 1988.

### Materials and methods

### *Subjects*

A total of nine liver specimens were obtained from patients of hepatocellular carcinoma at the time of operation at the department of surgery of Kagawa Medical School. Of the nine resected livers bearing hepatocellular carcinoma, three cancer tissues were not available for analysis because of the tissue necrosis. Five normal livers used as controls were obtained by liver biopsy for suspected disease during abdominal surgery, and had a normal histological appearance. Nine tissue regions distant from the cancer tissues in the resected livers with a normal histological appearance were also used as tissues of 'distant regions'. All liver tissues were immediately frozen in liquid nitrogen and stored at −80°C. The maximum time between removal of the biopsy sample and its being frozen did not exceed 20 min. These tissue samples were classified into three groups: cancer tissues, tissues of distant regions and normal tissues.

### *Chemicals*

IgG fractions of peroxidase-conjugated sheep anti-chicken IgG were purchased from Cappel Laboratories Inc. Bovine serum albumin was purchased from Boehringer Mannheim Inc. A protein assay kit was purchased from Bio-Rad Laboratories. Cellulose nitrate paper (pore size, 0.45 $\mu$m) was purchased from Toyo Roshi Co. Ltd. Acrylamide, *bis*-acrylamide and sodium dodecyl sulphate were purchased from Nakarai Chemicals Ltd. The other chemicals were purchased from Wako Pure Chemical Industries. These chemicals were of the highest quality commercially available.

Standard phospholipids were purchased from Serdary Research Laboratories. A cholesterol C-test kit was purchased from Wako Pure Chemical Industries.

### *Preparation of microsomes*

Liver tissues or cancer tissues were homogenised with a Teflon homogeniser in 0.25 M sucrose, and the pH was adjusted to 7.4 with 1.0 M Tris. The homogenates were centrifuged at 8,000 *g* for 10 min at 4°C. The supernatants were again centrifuged at 105,000 *g* for 60 min at 4°C. The precipitates were homogenised in 1.15% potassium chloride, pH 7.4, with a Teflon homogeniser and centrifuged at 105,000 *g* for 90 min to remove contaminating haemoglobin. The precipitates were stored at −80°C and used as microsomal pellets. The microsomes were suspended in 0.1 M

potassium phosphate buffer, pH 7.4, and were used as microsomal suspensions.

*Protein concentrations*

Protein concentrations were measured by the method of Bradford with a protein assay kit (Bradford, 1976).

*Enzyme preparations*

Cytochrome $b_5$, NADPH-cytochrome P-450 reductase and NADH-cytochrome $b_5$ reductase of bovine liver microsomes were purified by the methods of Spatz & Strittmatter (1973). These enzymes were electrophoretically pure and each gave a single protein band on SDS-polyacrylamide gel electrophoresis.

*SDS-polyacrylamide gel electrophoresis*

SDS-polyacrylamide gel electrophoresis was done by the method of Laemmli (1970). The purified cytochrome $b_5$, NADPH-cytochrome P-450 reductase and NADH-cytochrome $b_5$ reductase from bovine liver microsomes were measured spectrophotometrically with the molar extinction coefficients of 163.0 $mM^{-1} cm^{-1}$ at 424 nm (Spatz & Strittmatter, 1973), 21.4 $mM^{-1} cm^{-1}$ at 454 nm (Oprian & Coon, 1982), and 10.2 $mM^{-1} cm^{-1}$ at 460 nm (Spatz & Strittmatter, 1973), respectively.

*Antisera preparations*

Chicken anti-bovine cytochrome $b_5$, NADPH-cytochrome P-450 reductase and NADH-cytochrome $b_5$ reductase were prepared by the method of Hamamoto *et al.* (1986).

*Analytical procedures*

Microsomes were suspended in 0.1 M potassium phosphate buffer, pH 7.4 to the protein concentration of 1.0 $mg ml^{-1}$. The concentrations of cytochromes P-450 and P-420 of each suspension were measured from the difference spectra of sodium dithionite-reduced samples, with a value of 91 $mM^{-1} cm^{-1}$ assumed for the molar extinction increment between 450 and 490 nm for cytochrome P-450 (Omura & Sato, 1964*a*) and a value of 110 $mM^{-1} cm^{-1}$ assumed for between 420 and 490 nm for cytochrome P-420 (Omura & Sato, 1964*b*). In the measurement of the difference spectra, both sample and reference cuvets were bubbled with carbon monoxide to cancel the absorption peak derived from contaminated deoxyhaemoglobin, followed by reduction of the sample cuvet with sodium dithionite. By this method, the absorption of deoxyhaemoglobin can be cancelled, but methaemoglobin cannot be. To estimate the content of methaemoglobin in each tissue, the difference spectra were measured as follows: the sample and reference cuvets were bubbled with mixed gas of carbon monoxide and oxygen ($CO:O_2 = 1:1$ (v/v)) and the sample cuvet was reduced with sodium dithionite. In this difference spectrum, the absorption peak at 420 nm was that of methaemoglobin.

The concentrations of cytochrome $b_5$ in microsomal suspensions were measured by the absorbance difference of 424–409 nm in the difference spectra of NADH-reduced sample minus an air-saturated sample with the molar extinction coefficient 185 $mM^{-1} cm^{-1}$ (424–409 nm) by the method of Omura and Sato (1964*a*, *b*).

The cytochrome $b_5$, NADPH-cytochrome P-450 reductase and NADH-cytochrome $b_5$ reductase of the microsomes were measured by the Western blotting method followed by immunochemical staining by the method of Hamamoto *et al.* (1986).

Activities of NADPH- and NADH-ferricyanide and NADPH- and NADH-cytochrome c reductase of each microsome sample were measured by the method of Hiwatashi *et al.* (1976).

*Electron paramagnetic resonance (EPR) spectrum*

Portions of frozen tissues 2.2 mm across and 3 cm thick were punched out with a stainless steel tube. Each sample was put on the top of a quartz EPR tube with an inner diameter of 2.5 mm, which was filled with saline at room temperature and preintubed with a fine silicon tube connected with an injector (10 ml). Withdrawal of the piston moved the punched out sample to the bottom of the EPR tube in place of the saline. The EPR tube loaded with sample was frozen slowly from the bottom with liquid nitrogen and set in a JEOL Ltd EPR spectrophotometer. All EPR spectra were recorded in a first derivative display at a microwave power level below saturation. Electron paramagnetic resonance spectra were measured with a JEOL Ltd EPR spectrophotometer model JES-RE2X with an Air Products Ltd LTR-3-110 liquid helium cryostat and a JEOL Ltd data processor model ES-PRIT2.

*High-pressure liquid chromatography (HPLC)*

High-pressure liquid chromatography was done with a Toyo Soda liquid chromatographic system consisting of a Model CCPM pump and Model UV-8000 ultraviolet detector with a data processor Chromatocorder 11 from System Instruments Co. For phospholipid chromatography, the wavelength of the detector was set at 210 nm. Phospholipid separation was achieved with a Toyo Soda 4.6 × 250 mm column packed with TSK gel Silica-60 at room temperature. For routine phospholipid separations, the lipid extract was put on the column and a solvent system of acetonitrile–methanol–phosphate 900:95:5 (v/v/v) was used. The solvent mixture was delivered by the pump at a flow rate of 1.0 $ml min^{-1}$.

*Measurement of microsomal lipid composition*

*Phospholipid analysis* Lipid extraction was done by the method of Folch *et al.* (1957). Briefly, 0.1 ml of microsomal suspension containing 0.5 mg of protein was suspended in 0.9 ml of a 2:1 chloroform–methanol mixture (v/v) and thoroughly mixed with a vortex mixer for 3 min. The suspensions were centrifuged at 2,000 *g* for 10 min to remove the denatured proteins, and the supernatants were collected. These crude extracts were mixed thoroughly with 0.2 volumes of distilled water. The mixtures were centrifuged at 2,000 *g* for 10 min again. The upper phase was removed as quickly as possible, This washing procedure was repeated three times and the lower phases and the remaining rinsing fluids were put into one phase by the addition of 0.1 ml of methanol. These extracts were filtered with an Acro LC13 filter from Gelman Sciences Inc. Phospholipids in the solutions were analysed by HPLC by the method of Kaduce *et al.* (1983) with minor modifications.

*Cholesterol analysis* A microsome pellet (5 mg of protein) was suspended in 1 ml of 0.1 M potassium phosphate buffer, pH 7.4, containing 0.1% phenol and 0.3% cholic acid. This mixture was incubated for 30 min at room temperature. The cholesterol concentration of this mixture was measured with a Cholesterol C-test kit by the improved method of Barenholz *et al.* (1978).

### Results

*Concentrations of cytochromes b5, P-450 and P-420 in hepatocellular carcinoma*

The concentrations of cytochromes $b_5$, P-450 and P-420 of microsomes of human hepatocellular carcinoma and normal liver tissues were measured spectrophotometrically (Table I). These results indicate that the regions distant from the cancer nodules had cytochromes $b_5$, P-450 and P-420 at levels comparable to those in normal liver tissues. Even in

**Table I** Concentrations of cytochromes b5, P-450 and P-420 determined by spectrophotometry

| | *HCC* (*n*=6) | *Non-cancer region* (*n*=9) | *Normal liver* (*n*=5) |
|---|---|---|---|
| b5 (nmol per mg protein) | 0.018±0.026[a] | 0.127±0.047 | 0.145±0.023 |
| P-450 (nmol per mg protein) | 0.024±0.03[a] | 0.138±0.068 | 0.199±0.043 |
| P-420 (nmol per mg protein) | 0.106±0.056[a] | 0.030±0.048 | 0.018±0.019 |
| P-450+P-420 (nmol per mg protein) | 0.130±0.081 | 0.168±0.072 | 0.217±0.024 |
| P-450/(P-450+P-420) (%) | 14.0±12.3[a] | 84.7±21.6 | 91.0±9.74 |

HCC, hepatocellular carcinoma; ± indicates the standard deviation. [a]Statistically different from the values of normal liver at $P<0.01$.

**Table II** NADPH- and NADH-ferricyanide and cytochrome c reductase activities

| | *HCC* (*n*=6) | *Non-cancer region* (*n*=9) | *Normal liver* (*n*=5) |
|---|---|---|---|
| NADH-ferricyanide reductase activity (μmol per mg protein per min) | 1.12±0.32 | 1.39±0.21 | 1.60±0.071 |
| NADPH-ferricyanide reductase activity (μmol per mg protein per min) | 0.56±0.057 | 0.62±0.072 | 0.68±0.11 |
| NADH-cytochrome c reductase activity (nmol per mg protein per min) | 44.7±52.9[a] | 118.4±50.2 | 157.0±14.1 |
| NADPH-cytochrome c reductase activity (nmol per mg protein per min) | 10.5±8.6[a] | 27.3±39.0 | 33.2±8.3 |

HCC, hepatocellular carcinoma; ± indicates the standard deviation. [a]Statistically different from the values of normal liver at $P<0.01$.

the hepatocellular carcinoma tissue, the sum of cytochromes P-450 and P-420 are similar to those in normal livers or distant regions. But in hepatocellular carcinoma, the ratios of cytochrome P-450 to the sum of cytochromes P-450 and P-420 are smaller than for other samples. It is known that the affinity of haemoglobin to carbon monoxide is much stronger than that of cytochrome P-450 (Ichikawa *et al.*, 1967). In the measurement of the difference spectra using the mixed gas of carbon monoxide and oxygen, the purified cytochrome P-450 was not bound to carbon monoxide whereas haemoglobin was bound. Using this result, concentrations of contaminated methaemoglobin of the microsomal suspensions were measured and the contents of contaminated methaemoglobin were negligible compared to those of cytochrome P-420 (data not shown). So, the absorption peak at 420 nm of the carbon monoxide-difference spectrum was looked upon as that of cytochrome P-420.

The concentrations of cytochrome $b_5$ in the cancer tissues were also decreased to about one-eighth those in normal liver tissues or distant regions of the cancers.

*Specific activities of NADPH- or NADH-ferricyanide reductase, cytochrome c reductase and cytochrome P-450 reductase*

Table II shows the activities of NADPH- and NADH-ferricyanide reductase and NADPH- and NADH-cytochrome c reductase of the microsomal preparations. The results indicate that the activities of NADPH- or NADH-ferricyanide reductase were almost the same for the three groups. On the other hand, NADPH- or NADH-cytochrome c reductase activities were higher in normal livers or distant regions than in hepatocellular carcinoma tissues. These findings suggest that in microsomes from the cancer, the electron can be easily transferred to ferricyanide, but it is difficult for macromolecules such as cytochrome c.

*Measurement of cytochrome $b_5$, NADPH-cytochrome $b_5$ reductase and NADPH-cytochrome P-450 reductase by Western blotting*

NADPH- and NADH-cytochrome c reductase activities were low in hepatocellular carcinoma tissues for two possible reasons: a decrease in the concentrations of cytochrome $b_5$, NADPH-cytochrome P-450 reductase or NADH-cytochrome $b_5$ reductase in cancer tissues, or low efficiency of electron transport from NADH or NADPH to cytochrome c via NADH-cytochrome $b_5$ reductase. The concentrations of cytochrome $b_5$, NADH-cytochrome $b_5$ reductase and NADPH-cytochrome P-450 reductase were measured immunochemically. Figure 1 shows the immunochemically stained cellulose nitrate papers of typical tissues stained with antibodies of cytochrome $b_5$, NADH-cytochrome $b_5$ reductase and NADPH-cytochrome P-450 reductase. The results are summarised in Table III, which shows that the concentrations and the molecular weights of the three components are almost the same for all microsomal samples. This supports the second possibility.

**Figure 1** Enzyme bands of the typical cancer tissues on cellulose nitrate papers stained with antibodies to cytochrome $b_5$, NADH-cytochrome b5 reductase and NADPH-cytochrome P-450 reductase, respectively. Each well contained 0.5 mg of proteins of microsomes of cancer and normal tissues. (a) Stained with rabbit anti-bovine cytochrome b5; (b) stained with chicken anti-bovine NADH-cytochrome b5 reductase; (c) stained with chicken anti-bovine NADPH-cytochrome P-450 reductase. 1,2, normal liver tissues; 3–5, hepatocellular carcinoma tissues.

**Table III** Concentrations of cytochrome $b_5$, NADH-cytochrome $b_5$ reductase and NADPH-cytochrome P-450 reductase by Western blotting method followed by immunochemical staining

| | *HCC* (*n*=*6*) | *Non-cancer region* (*n*=*9*) | *Normal liver* (*n*=*5*) |
|---|---|---|---|
| $b_5$ (pmol per protein) | 14.1±6.3 | 24.0±8.5 | 11.6±4.3 |
| $b_5$ reductase (pmol per mg protein) | 19.4±10.5 | 28.2±7.8 | 28.1±12.3 |
| P-450 reductase (pmol per mg protein) | 120.2±42.8 | 133.0±46.2 | 187.9±56.2 |

HCC, hepatocellular carcinoma, ± indicates the standard deviation.

**Table IV** Phospholipids and cholesterol compositions measured by high pressure liquid chromatography

| | *HCC* (*n*=*6*) | *Non-cancer region* (*n*=*9*) | *Normal liver* (*n*=*5*) |
|---|---|---|---|
| Phospholipids (μg per mg protein) | 89.8±36.1 | 104.1±34.2 | 105.5±3.7 |
| Phosphatidyl choline | 27.0±9.7[a] | 46.4±20.6 | 51.9±13.9 |
| Phosphatidyl ethanolamine | 19.3±11.8 | 27.1±11.9 | 31.0±14.3 |
| Phosphatidyl inositol | 15.3±2.63 | 14.8±4.0 | 17.3±3.31 |
| Phosphatidyl serine | 28.3±24.8 | 15.7±12.0 | 5.38±0.01 |
| Cholesterols (μg per mg protein) | 7.08±3.22[a] | 4.64±1.39[a] | 2.49±0.48 |
| Phospholipid/cholesterol | 13.1±3.13[a] | 23.1±8.42[a] | 43.0±6.74 |

HCC, hepatocellular carcinoma; ± indicates the standard deviation. [a]Statistically different from the values of normal liver at $P<0.05$.

*Lipid compositions of the microsomes*

The lipid compositions of the prepared microsomes of normal and cancer liver tissues were analysed by HPLC. Phospholipid compositions, cholesterol concentrations and phospholipid/cholesterol ratios of microsomes of the three groups are listed in Table IV. This table indicates that phosphatidyl choline was decreased and cholesterols were increased in the cancer tissues compared with the tissues of distant region or normal liver, so the phospholipid/cholesterol ratios were decreased to 13.1 (w/w) in cancer microsomes. In the distant region from the cancer nodule, only cholesterols were increased and the phospholipid/cholesterol ratios were slightly decreased.

### Discussion

The cytochrome P-450-linked monooxygenase systems of human hepatocellular carcinoma were investigated optically and compared with normal liver tissues. In hepatocellular carcinoma, the sum of microsomal cytochromes P-450 and P-420 per millgram of protein were almost the same as those of the distant regions or normal liver tissues, but the ratios of cytochrome P-450 to the sum of cytochrome P-450 and P-420 were much lower in the microsomes of the hepatocellular carcinoma tissues than in normal liver microsomes, although the microsomes from the cancer tissue and its distal region were simultaneously prepared. The stability of cytochrome P-450 molecules in cancer tiisues may be affected by the abnormal protein structure of an abnormality of the microsomal membranes.

In order to observe the instability of cytochrome P-450 molecules in whole tissues of the cancer, distant region and normal liver, the electron paramagnetic resonance spectra were measured at 15 K; typical spectra are shown in Figure 2. Judging from the signal height at $g=2.25$ for low spin form and $g=8.05$ for high spin form, the cytochrome P-450 contents of whole tissues were decreased to about half in hepatocellular carcinoma tissues compared to the normal liver tissues. These differences in cytochrome P-450 contents in normal and cancer tissues may be due to the changes of circumstances of cytochrome P-450 molecules in cancer tissues, because the sum of the concentrations of cytochromes P-450 and P-420 in cancer tissues is almost comparable to that in the normal tissues spectrophotometrically. The term 'circumstances' means the lipid composition of the

**Figure 2** EPR spectra of human liver tissues of normal and cancerous regions. The spectra were measured at 15 K. Microwave power, 5 mW; modulation amplitude, 10 G; scan speed, 625 G min$^{-1}$.

microsomal membrane, endogenous substrate, pH, microsomal membrane fluidity and electrolyte composition. Some kinds of hepatocellular carcinoma tissues had a strong EPR signal at $g=6.70$ and weak EPR cytochrome P-450 signals at other $g$ values. This spectrum indicates that in certain kinds of hepatocellular carcinoma, the rhombicity of the haem moiety of cytochrome P-450 molecule may be changed; in such cancer tissue, almost all cytochrome P-450 is irreversibly converted to cytochrome P-420. These results strongly suggest that cytochrome P-450 of the cancer tissues is already converted to the denatured form 'cytochrome P-420' even in the intact cancer cells, although cytochrome P-420 gives various spin forms (Ichikawa & Yamano, 1970).

Activities of NADPH- and NADH-ferricyanide reductase and NADPH- and NADH-cytochrome c reductase of each kind of microsome were measured. In cancer microsomes, ferricyanide reductase activities were the same as those in normal microsomes, but cytochrome c reductase activities were low. These findings suggest that in cancer microsomes the electron can be easily transferred by the microsomal cytochrome P-450-linked electron transport systems in hepatocellular carcinoma tissues to smaller molecules such as ferricyanide, but not to cytochrome c, and that the electron transport efficiency is low. Thus, in the microsomal cytochrome P-450-linked monooxygenase system, the electron transport from NADH or NADPH to cytochrome P-450 is impaired.

This property of microsomal cytochrome P-450-linked monooxygenase systems in hepatocellular carcinoma tissues may be explained by the following hypotheses: (1) abnormal protein structure of the components of the monooxygenase system; (2) aponisation of the components of cytochrome P-450-linked monooxygenase system; and (3) abnormal structures and contents of the components of the microsomal membranes.

The microsomes were analysed by the Western blotting method with antibodies to cytochrome $b_5$, NADH-cytochrome $b_5$ reductase or NADPH-cytochrome P-450 reductase. The concentrations were at the same levels in normal and cancer microsomes. Their monomeric molecular weights were the same. This indicates that in the cancer microsomes apoenzymes of the microsomal monooxygenase systems that cross-react with each antibody exist in equal amounts. The concentration of cytochrome $b_5$ measured by Western blotting was less than the optical concentration. This may be due to the affinity of the immune reaction of the antibodies used in this experiment to human microsomal cytochrome $b_5$ being lower than its affinity to that of bovine microsomes with which the antibodies were made. The concentrations of NADPH-cytochrome P-450 reductase and NADH-cytochrome $b_5$ reductase measured by Western blotting may also be lower than the real concentrations.

To examine hypothesis 3, the lipid compositions of each kind of microsome were measured and the phospholipid/cholesterol ratios were calculated. The concentrations of cholesterols per milligram of protein were increased and those of phosphatidyl choline were decreased in cancer microsomes, but the total phospholipid concentration was not different. The cancer microsomes had lower phospholipid/cholesterol ratios than those of other microsomes, and the microviscosity of the cancer microsomes may be lower than that of normal microsomes (Kapitulnik et al., 1987). Some membrane-bound enzymes, such as glucose-6-phosphatase, UDP-glucuronyltransferase and calcium-dependent ATPase, are unstable after treatment with phospholipase A (Fiehn & Hasselbach, 1970). Strobel et al. (1970) reported that the interaction between NADPH-cytochrome P-450 reductase and cytochrome P-450 requires the presence of phosphatidyl choline. The microsomal cytochrome P-450-linked monooxygenase systems may require the normal composition of phospholipids, especially phosphatidyl choline, and cholesterols to transport electrons efficiently. These results strongly support possibility 3 for the low efficiency of electron transport in cancer microsomes.

In primary cancer of the mammalian liver, the activities of both oxidative (phase I) and conjugative (phase II) enzymes are decreased (El Mouelhi et al., 1987). In human liver malignancies such as hepatocellular carcinoma, it seems that the membrane lipid composition is different from that of normal liver tissue. The difference may reduce the oxidative and conjugative activities of the cancer.

The biochemical properties, including the concentrations and activities of microsomal cytochrome P-450-linked monooxygenase systems and microsomal lipid compositions of the distant regions were not statistically different from those of normal liver tissues except for the cholesterol concentrations. Whether this difference has some meaning or not remains to be investigated.

### References

ASTROM, A., DEPIERRE, J.W. & ERIKSSON, L. (1983). Characterization of drug metabolizing systems in hyperplastic nodules from the livers of rats receiving 2-acetylaminofluorene in their diet. *Carcinogenesis*, **4**, 577.

BARENHOLZ, Y., PATZER, E.J., MOOR, N.F. & WAGNER, R.R. (1978). Cholesterol oxidase as a probe for studying membrane composition and organization. In *Enzymes of Lipid Metabolism*, Gatt, S., Freysz, L. & Mandel, P. (eds) p. 45. Plenum Press: New York.

BENGA, G., POP, V.I., IONESCU, M., HODARNAU, A., TILINCA, R. & FRANGOPOL, P.T. (1983). Composition of human and rat liver microsomes by spin label and biochemical analyses. *Biochim. Biophys. Acta*, **750**, 194.

BRADFORD, M.M. (1976). A rapid and sensitive method for the quantitation of microgram quantities of protein utilizing the principle of protein-dye binding. *Anal. Biochem.*, **72**, 248.

COOPER, D.Y., LEVIN, S., NARASIMHULU, S., ROSENTHAL, O. & ESTABROOK, R.W. (1965). Photochemical action spectrum of the terminal oxidase of mixed function oxidase systems. *Science*, **147**, 400.

EL MOUELHI, M., DIDOLKAR, M.S., ELIAS, E.G., GUENGERICH, F.P. & KAUFFMAN, F.C. (1987). Hepatic drug-metabolizing enzymes in primary and secondary tumors of human liver. *Cancer Res.*, **47**, 460.

ERIKSSON, L.C., TORNDAL, U.B. & ANDERSSON, G.N. (1983). Isolation and characterization of endoplasmic reticulum and Golgi apparatus from hepatocyte nodules in male Wistar rats. *Cancer Res.*, **43**, 3335.

FARBE, E. (1984). The biochemistry of preneoplastic liver: A common metabolic pattern in hepatocyte nodules. *Can. J. Biochem. Cell Biol.*, **62**, 486.

FIEHN, W. & HASSELBACH, W. (1970). The effect of phospholipase A on the calcium transport and the role of unsaturated fatty acids in ATPase activity of sarcoplasmic vesicles. *Eur. J. Biochem.*, **13**, 510.

FOLCH, J., LEES, M. & STANLEY, S. (1957). A simple method for the isolation and purification of total lipids from animal tissues. *J. Biol. Chem.*, **226**, 497.

HAMAMOTO, I., HIWATASHI, A. & ICHIKAWA, Y. (1986). Zonal distribution of cytochrome P-450 and related enzymes of bovine adrenal cortex – quantitative assay of concentrations and total contents. *J. Biochem.*, **99**, 1743.

HIWATASHI, A., ICHIKAWA, Y., MARUYA, N., YAMANO, T. & AKI, K. (1976). Properties of crystalline reduced nicotinamide adenine dinucleotide phosphate-adrenodoxin reductase from bovine adrenocortical mitochondria. I. Physiochemical properties of holo- and apo-NADPH-adrenodoxin reductase and interaction between non-heme iron proteins and the reductase. *Biochemistry*, **15**, 3082.

HRYCAY, E.G. & PROUGH, R.A. (1974). Reduced nicotinamide adenine dinucleotide-cytochrome $b_5$ reductase and cytochrome $b_5$ as electron carriers in NADH-supported cytochrome P-450-dependent enzyme activities in liver microsomes. *Arch. Biochem. Biophys.*, **165**, 331.

ICHIKAWA, Y. & YAMANO, T. (1970). Preparation and physicochemical properties of functional hemoprotein P-450 from mammalian tissue microsomes. *Biochim. Biophys. Acta*, **200**, 220.

ICHIKAWA, Y., HAGIHARA, B. & YAMANO, T. (1967). Magnetic and spectrophotometric properties of the microsomal CO-binding pigment. *Arch. Biochem. Biophys.*, **120**, 204.

IYANAGI, T. & MASON, H.S. (1973). Some properties of hepatic reduced nicotinamide adenine dinucleotide phosphate-cytochrome c reductase. *Biochemistry*, **12**, 2297.

KADUCE, T.L., NORTON, K.C. & SPECTOR, A.A. (1983). A rapid, isocratic method for phospholipid separation by high-performance liquid chromatography. *J. Lipid. Res.*, **24**, 1398.

KAPITULNIK, J., WEIL, E., RABINOWITZ, R. & KRAUSZ, M.M. (1987). Fetal and adult human liver differ markedly in the fluidity and lipid composition of their microsomal membranes. *Hepatology*, **7**, 55.

LAEMMLI, U.K. (1970). Cleavage of structural proteins during the assembly of the head of bacteriophage T4. *Nature*, **227**, 680.

MASON, H.S., YAMANO, T., NORTH, J.C., HASHIMOTO, Y. & SAKAGISHI, P. (1965). In *Oxidases and Related Redox Systems, Vol. 2*, King, T., Mason, H.S. & Morrison, M. (eds) p. 879. Wiley: Chichester.

NORMAN, R.L., MULLER-EBERHARD, U. & JOHNSON, E.F. (1979). The role of cytochrome P-450 forms in 2-aminoanthracene and benzo(a)pyrene mutagenesis. *Biochem. Biophys. Res. Commun.*, **89**, 195.

OMURA, T. & SATO, R. (1964*a*). The carbon monoxide-binding pigment of liver microsomes. *J. Biol. Chem.*, **239**, 2370.

OMURA, T. & SATO, R. (1964*b*). The carbon monoxide-binding pigment of liver microsomes. *J. Biol. Chem.*, **239**, 2379.

OPRIAN, D.D. & COON, M.J. (1982). Oxidation-reduction states of FMN and FAD in NADPH-cytochrome P-450 reductase during reduction by NADPH. *J. Biol. Chem.*, **257**, 8935.

SPATZ, L. & STRITTMATTER, P. (1973). A form of reduced nicotinamide adenine dinucleotide-cytochrome $b_5$ reductase containing both the catalytic site and an additional hydrophobic membrane-binding segment. *J. Biol. Chem.*, **248**, 793.

STROBEL, H.W., LU, A.Y., HEIDEMA, J. & COON, M.J. (1970). Phosphatidylcholine requirement in the enzymatic reduction of hemoprotein P-450 and in fatty acid, hydrocarbon, and drug hydroxylation. *J. Biol. Chem.*, **245**, 4851.

WANG, P.P., BEAUNE, P., KAMINSKY, L.S. & 4 others (1983). Purification and characterization of six cytochrome P-450 isoenzymes from human liver microsomes. *Biochemistry*, **22**, 5375.

WASKELL, L., KOBLIN, D. & CANOVA-DAVIS, E. (1982). The lipid composition of human liver microsomes. *Lipids*, **17**, 317.

WRIGHTON, S.A., CAMPANILE, C., THOMAS, P.E. & 8 others (1986). Identification of a human liver cytochrome P-450 homologous to the major isosafrole-inducible cytochrome P-450 in the rat. *Mol. Pharmacol.*, **29**, 405.


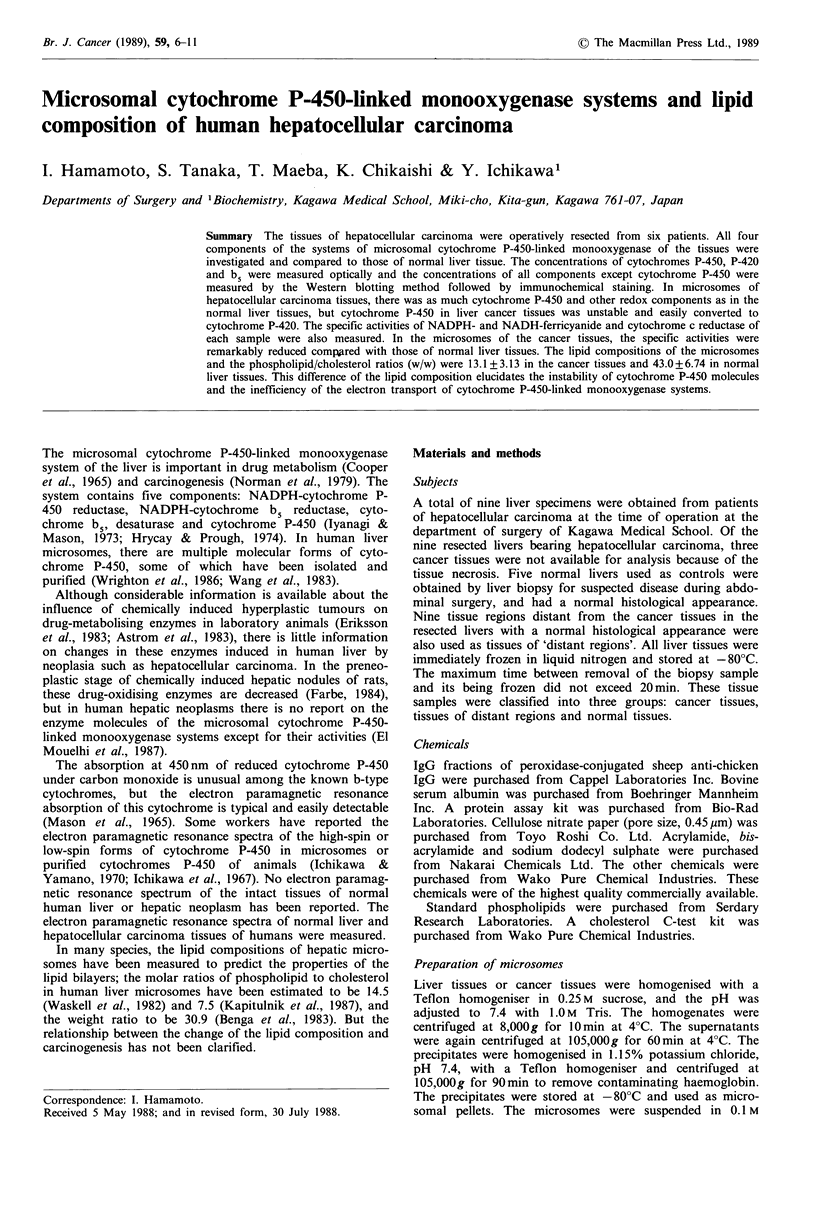

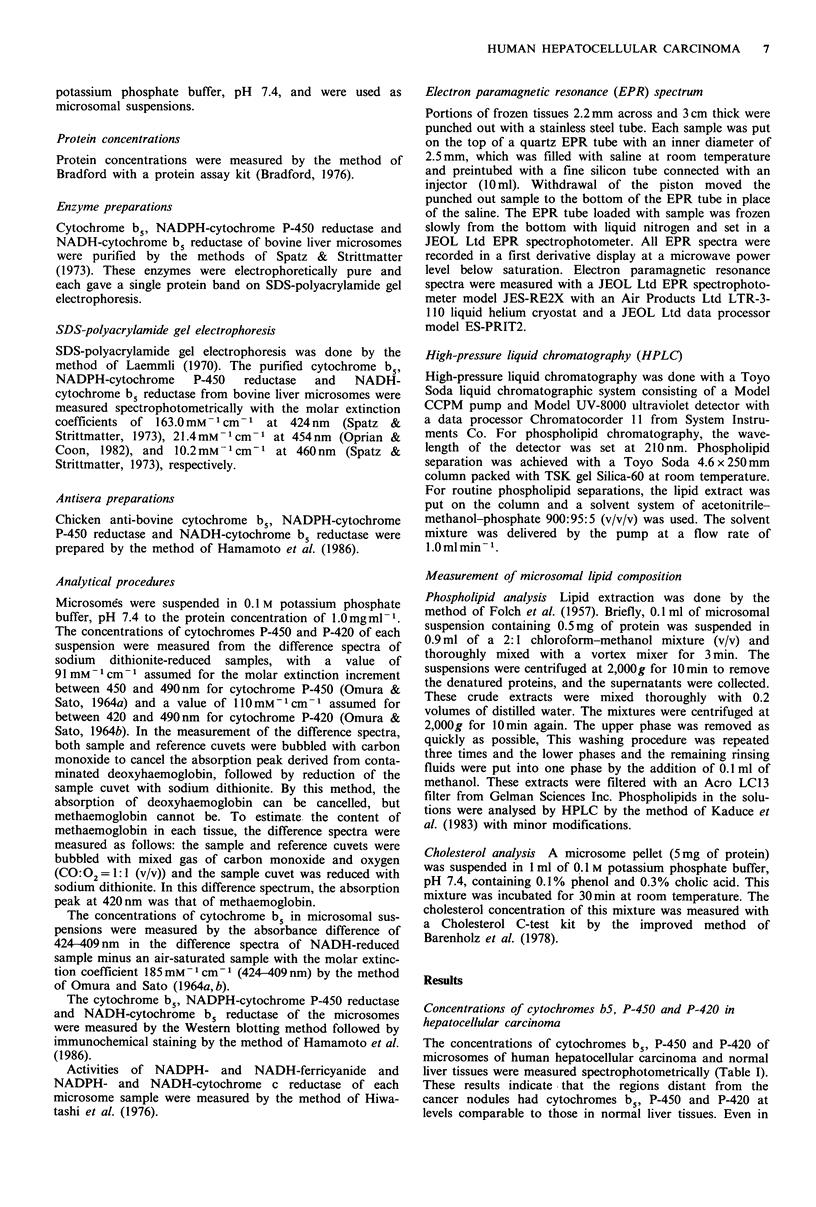

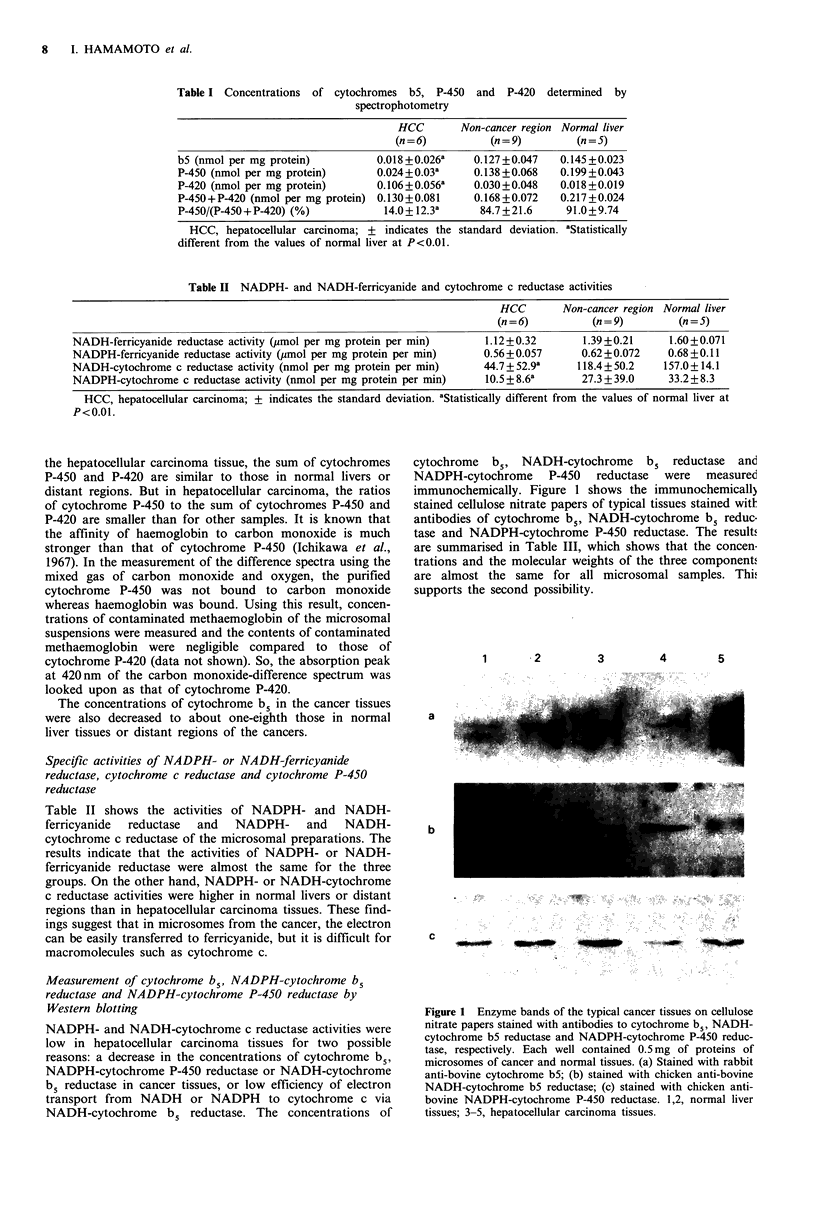

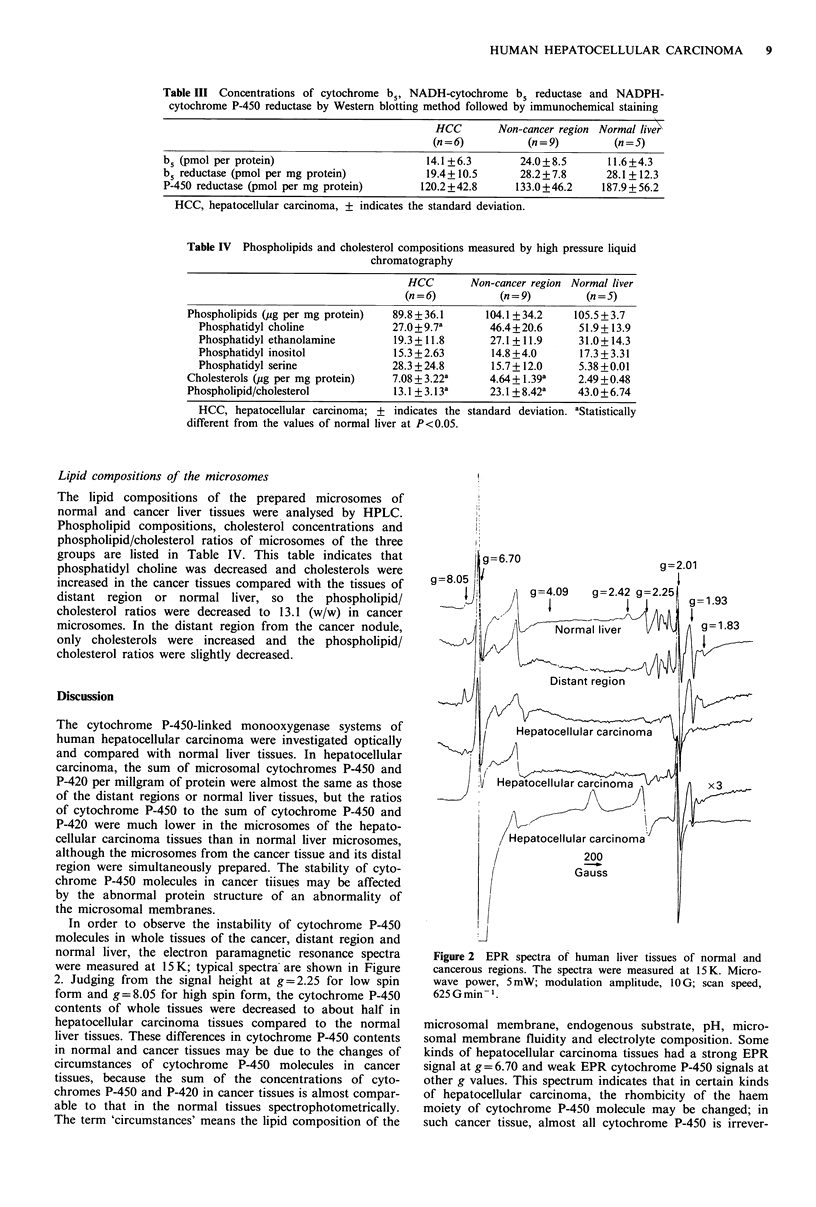

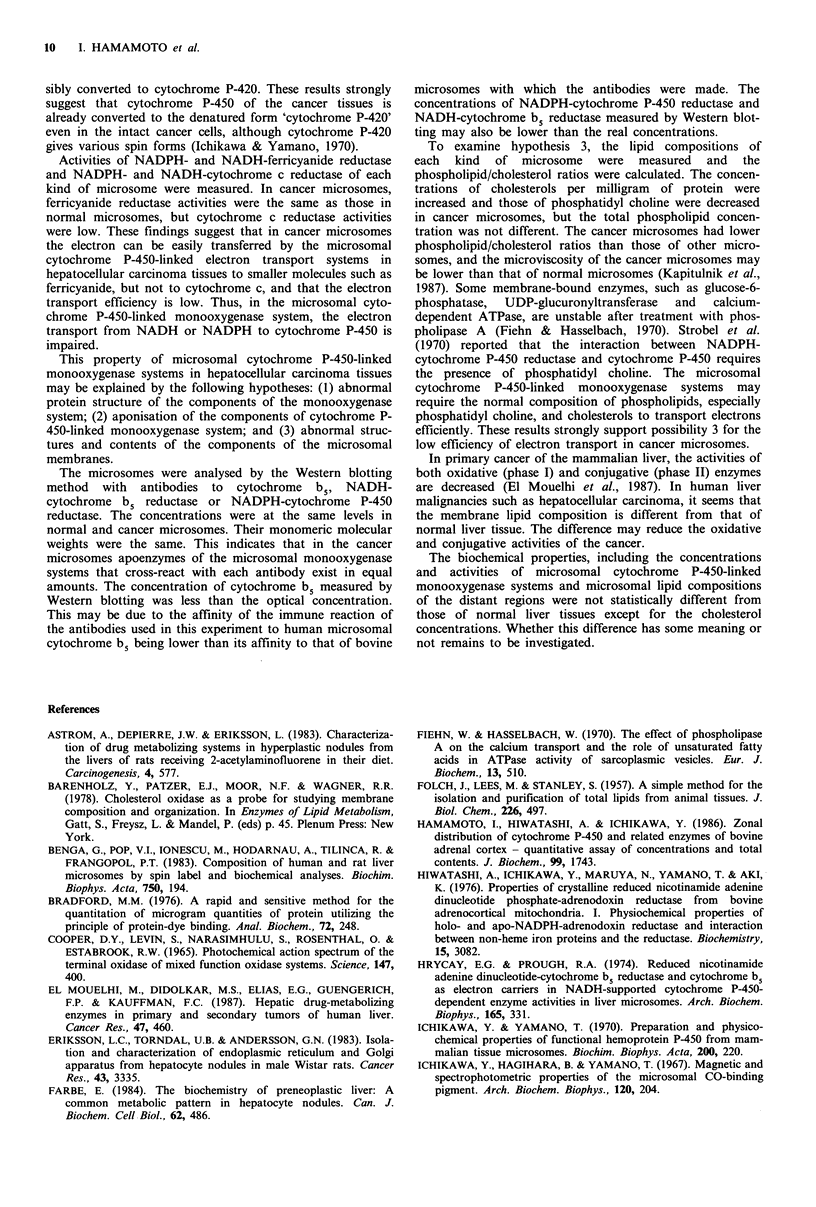

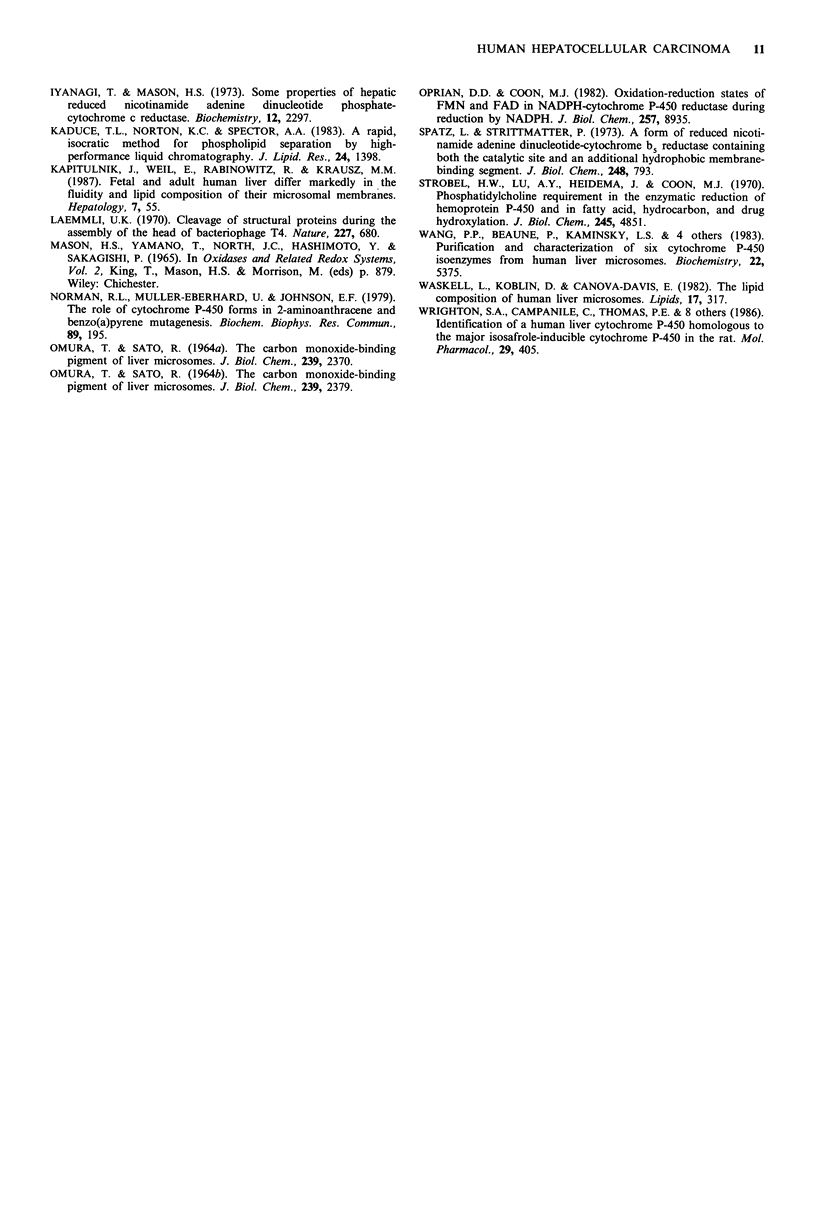


## References

[OCR_00589] Aström A., DePierre J. W., Eriksson L. (1983). Characterization of drug-metabolizing systems in hyperplastic nodules from the livers of rats receiving 2-acetylaminofluorene in their diet.. Carcinogenesis.

[OCR_00602] Benga G., Pop V. I., Ionescu M., Hodârnău A., Tilinca R., Frangopol P. T. (1983). Comparison of human and rat liver microsomes by spin label and biochemical analyses.. Biochim Biophys Acta.

[OCR_00608] Bradford M. M. (1976). A rapid and sensitive method for the quantitation of microgram quantities of protein utilizing the principle of protein-dye binding.. Anal Biochem.

[OCR_00613] COOPER D. Y., LEVIN S., NARASIMHULU S., ROSENTHAL O. (1965). PHOTOCHEMICAL ACTION SPECTRUM OF THE TERMINAL OXIDASE OF MIXED FUNCTION OXIDASE SYSTEMS.. Science.

[OCR_00625] Eriksson L. C., Torndal U. B., Andersson G. N. (1983). Isolation and characterization of endoplasmic reticulum and Golgi apparatus from hepatocyte nodules in male wistar rats.. Cancer Res.

[OCR_00642] FOLCH J., LEES M., SLOANE STANLEY G. H. (1957). A simple method for the isolation and purification of total lipides from animal tissues.. J Biol Chem.

[OCR_00631] Farber E. (1984). The biochemistry of preneoplastic liver: a common metabolic pattern in hepatocyte nodules.. Can J Biochem Cell Biol.

[OCR_00636] Fiehn W., Hasselbach W. (1970). The effect of phospholipase A on the calcium transport and the role of unsaturated fatty acids in ATPase activity of sarcoplasmic vesicles.. Eur J Biochem.

[OCR_00647] Hamamoto I., Hiwatashi A., Ichikawa Y. (1986). Zonal distribution of cytochromes P-450 and related enzymes of bovine adrenal cortex--quantitative assay of concentrations and total contents.. J Biochem.

[OCR_00653] Hiwatashi A., Ichikawa Y., Maruya N., Yamano T., Aki K. (1976). Properties of crystalline reduced nicotinamide adenine dinucleotide phosphate-adrenodoxin reductase from bovine adrenocortical mitochonria. I. Physicochemical properties of holo- and apo-NADPH-adrenodoxin reductase and interaction between non-heme iron proteins and the reductase.. Biochemistry.

[OCR_00662] Hrycay E. G., Prough R. A. (1974). Reduced nicotinamide adenine dinucleotide-cytochrome b5 reductase and cytochrome b5 as electron carriers in NADH-supported cytochrome P-450 -dependent enzyme activities in liver microsomes.. Arch Biochem Biophys.

[OCR_00674] Ichikawa Y., Hagihara B., Yamano T. (1967). Magnetic and spectrophotometric properties of the microsomal carbon monoxide binding pigment.. Arch Biochem Biophys.

[OCR_00669] Ichikawa Y., Yamano T. (1970). Preparation and physicochemical properties of functional hemoprotein P450 from mammalian tissue microsomes.. Biochim Biophys Acta.

[OCR_00681] Iyanagi T., Mason H. S. (1973). Some properties of hepatic reduced nicotinamide adenine dinucleotide phosphate-cytochrome c reductase.. Biochemistry.

[OCR_00686] Kaduce T. L., Norton K. C., Spector A. A. (1983). A rapid, isocratic method for phospholipid separation by high-performance liquid chromatography.. J Lipid Res.

[OCR_00691] Kapitulnik J., Weil E., Rabinowitz R., Krausz M. M. (1987). Fetal and adult human liver differ markedly in the fluidity and lipid composition of their microsomal membranes.. Hepatology.

[OCR_00697] Laemmli U. K. (1970). Cleavage of structural proteins during the assembly of the head of bacteriophage T4.. Nature.

[OCR_00707] Norman R. L., Muller-Eberhard U., Johnson E. F. (1979). The role of cytochrome P-450 forms in 2-aminoanthracene and benz[alpha]pyrene mutagenesis.. Biochem Biophys Res Commun.

[OCR_00713] OMURA T., SATO R. (1964). THE CARBON MONOXIDE-BINDING PIGMENT OF LIVER MICROSOMES. I. EVIDENCE FOR ITS HEMOPROTEIN NATURE.. J Biol Chem.

[OCR_00717] OMURA T., SATO R. (1964). THE CARBON MONOXIDE-BINDING PIGMENT OF LIVER MICROSOMES. II. SOLUBILIZATION, PURIFICATION, AND PROPERTIES.. J Biol Chem.

[OCR_00721] Oprian D. D., Coon M. J. (1982). Oxidation-reduction states of FMN and FAD in NADPH-cytochrome P-450 reductase during reduction by NADPH.. J Biol Chem.

[OCR_00726] Spatz L., Strittmatter P. (1973). A form of reduced nicotinamide adenine dinucleotide-cytochrome b 5 reductase containing both the catalytic site and an additional hydrophobic membrane-binding segment.. J Biol Chem.

[OCR_00732] Strobel H. W., Lu A. Y., Heidema J., Coon M. J. (1970). Phosphatidylcholine requirement in the enzymatic reduction of hemoprotein P-450 and in fatty acid, hydrocarbon, and drug hydroxylation.. J Biol Chem.

[OCR_00738] Wang P. P., Beaune P., Kaminsky L. S., Dannan G. A., Kadlubar F. F., Larrey D., Guengerich F. P. (1983). Purification and characterization of six cytochrome P-450 isozymes from human liver microsomes.. Biochemistry.

[OCR_00744] Waskell L., Koblin D., Canova-Davis E. (1982). The lipid composition of human liver microsomes.. Lipids.

[OCR_00748] Wrighton S. A., Campanile C., Thomas P. E., Maines S. L., Watkins P. B., Parker G., Mendez-Picon G., Haniu M., Shively J. E., Levin W. (1986). Identification of a human liver cytochrome P-450 homologous to the major isosafrole-inducible cytochrome P-450 in the rat.. Mol Pharmacol.

[OCR_00619] el Mouelhi M., Didolkar M. S., Elias E. G., Guengerich F. P., Kauffman F. C. (1987). Hepatic drug-metabolizing enzymes in primary and secondary tumors of human liver.. Cancer Res.

